# 

**Published:** 1887-05

**Authors:** 


Whole Number, 329 MAY, 1887. Vol. LIV., Number 5

LIBRARY INDEXED S.G.O.

THE

### CHICAGO MEDICAL JOURNAL AND EXAMINER

Editor: S. J. JONES, M.D., LL.D.

PUBLISHED MONTHLY BY

THE CLARK & LONGLEY COMPANY,

Under direction of

THE CHICAGO MEDICAL PRESS ASSOCIATION,

308-316 DEARBORN STREET.

